# Bioactive Polyphenols from Pomegranate Juice Reduce 5-Fluorouracil-Induced Intestinal Mucositis in Intestinal Epithelial Cells

**DOI:** 10.3390/antiox9080699

**Published:** 2020-08-03

**Authors:** Giacomo Pepe, Shara Francesca Rapa, Emanuela Salviati, Alessia Bertamino, Giulia Auriemma, Stella Cascioferro, Giuseppina Autore, Andrea Quaroni, Pietro Campiglia, Stefania Marzocco

**Affiliations:** 1 Department of Pharmacy, University of Salerno, I-84084 Salerno, Italy; gipepe@unisa.it (G.P.); srapa@unisa.it (S.F.R.); esalviati@unisa.it (E.S.); abertamino@unisa.it (A.B.); gauriemma@unisa.it (G.A.); autore@unisa.it (G.A.); 2 PhD Program in Drug Discovery and Development, University of Salerno, I-84084 Salerno, Italy; 3 Dipartimento di Scienze e Tecnologie Biologiche, Chimiche e Farmaceutiche (STEBICEF), University of Palermo, I-90128 Palermo, Italy; stellamaria.cascioferro@unipa.it; 4 Department of Biomedical Sciences, Cornell University, Veterinary Research Tower, Ithaca, NY 14853-6401, USA; aq10@cornell.edu; 5 European Biomedical Research Institute of Salerno, I-84125 Salerno, Italy

**Keywords:** intestinal epithelial cells, *Punica granatum* L., inflammation, oxidative stress, 5-fluorouracil, gastrointestinal mucositis, polyphenols, onconutraceutical

## Abstract

Intestinal epithelial cells (IECs) play a pivotal role in maintaining intestinal homeostasis. Different noxious agents, among them also anticancer therapies, can impair intestinal epithelial integrity triggering inflammation and oxidative stress. A frequent complication of chemotherapy is gastrointestinal mucositis, strongly influencing the effectiveness of therapy, increasing healthcare costs, and impairing patients’ quality of life. Different strategies are used to treat gastrointestinal mucositis, including products from natural sources. Our study focused on the effect of pomegranate (*Punica granatum* L.) juice extract on IEC-6 cells, both during inflammatory conditions and following treatment with 5-fluorouracil (5-FU). The polyphenolic profile of pomegranate juice was characterized in detail by Online Comprehensive two dimensional Liquid Chromatography-Mass Spectrometry. The evaluation of pomegranate juice extract in IEC-6 indicates a significant inhibition in proinflammatory factors, such as cytokines release, cyclooxygenase-2 and inducible nitric oxide synthase expression, and nitrotyrosine formation. Pomegranate also inhibited oxidative stress and adhesion protein expression. In 5-FU-treated IEC-6, pomegranate also inhibited both inflammatory and oxidative stress parameters and apoptosis. It promoted wound repair and tight junction expression. These results suggest a potential use of pomegranate as an adjuvant in the treatment of intestinal inflammatory and oxidative stress states, which also occur during chemotherapy-induced mucositis.

## 1. Introduction

The gut is a complex organ playing a key role in nutrients absorption and tolerance for harmless/beneficial microorganisms, while maintaining the ability to defend itself from pathogenic microorganisms [1]. However, actors such as infections, host damage and danger signaling can induce a change in intestinal homeostasis, leading to inflammation, which aims to restore normal homeostatic levels and organ function. [2]. Multiple environmental factors have been identified as potential triggers of uncontrolled intestinal inflammatory conditions such as in inflammatory bowel diseases (IBDs) and uncontrolled inflammatory pathologies of the gastrointestinal tract. In particular, intestinal epithelial cells (IECs) can perceive and respond to microbial or external stimuli to strengthen barrier function and participate in an adequate immune response [3] also associated with an imbalance in the body’s redox defense systems, giving rise to oxidative stress, which is presently considered as potentially critical in the pathogenesis, progression, and severity of IBDs [4].

Both inflammation and oxidative stress are also linked as side effects at the gastrointestinal level (mucositis) of chemotherapy (CT) and radiotherapy (RT). This side effect occurs in 20–40% of patients receiving CT for solid tumors, 60–80% of those undergoing hematopoietic stem cell transplantation and in almost all patients receiving RT for head and neck cancers [5,6,7]. Mucositis is a very complex process that leads to inflammatory and/or ulcerative lesions, which can affect any part of the gastrointestinal tract, damaging the mucosa. The specific mechanism of mucositis, along with its clinical presentation, depends on the anatomical site involved (oral or gastrointestinal) and on the type of CT and RT [8,9].

Gastrointestinal mucositis is often responsible for nausea, vomiting and diarrhea. The onset, duration and clinical presentation of this complication are also affected by patient-related risk factors, including age, ethnicity, gender and other disorders such as malnutrition that may increase the risk of developing mucositis [10,11]. Mucositis can have a serious impact on the patient’s quality of life and can negatively affect the ability to properly adhere to chemotherapy treatment. In addition, it has a non-negligible economic effect due to the cost of care [12]. The correct classification of the lesions is fundamental for the choice of the most effective pharmacological treatment. The therapeutic success and quality of life of the cancer patient also depend on an early diagnosis and management of the complications of anticancer therapy. Nowadays, different strategies are used to treat mucositis and, among them also products from natural sources, which possess anti-apoptotic, anti-inflammatory and antioxidant properties [13,14,15].

Pomegranate (*Punica granatum* L.) is one of the first domesticated fruits that has been cultivated from past times. Pomegranate fruits have been used to expel parasites [16], seeds and fruit peels to treat diarrhea [17,18], flowers to manage diabetes [19], tree barks and roots to stop bleeding and heal ulcers, and leaves to control inflammation and treat digestive system disorders [20]. Moreover, the ingredients of pomegranate show a chemopreventive role through the inactivation and activation of cell signaling pathways including tumor suppressor genes, angiogenesis and apoptotic pathways [21,22]. Due to all of these positive effects for human health, pomegranate has attracted great interest in recent years [23].

Considering the properties of polyphenols present in pomegranate juice extract (PPJE), in this study we evaluated its effect on inflammatory and oxidative stress mediators in IECs both during inflammatory response and 5-Fluorouracil (5-FU) treatment.

## 2. Materials and Methods

### 2.1. Reagents and Standards

Ultra pure water (H_2_O) was from a Milli-Q Direct 8 system (Millipore, Milan, Italy). Unless otherwise specified, all reagents and compounds were purchased from Sigma Chemicals Company (Sigma, Milan, Italy).

### 2.2. Sample Preparation

*Punica granatum* L. (pomegranate) fruits were purchased in a local store in Fisciano (SA, Campania, Italy). They were manually washed and peeled, then the arils were squeezed with a commercial electric homogenizer. In order to remove the pulp, the juice obtained was centrifuged at 6000 rpm, 4 °C, for 10 min (Mikro 220R× *g*, Hettich, Germany), and then the supernatant was lyophilized for 72 h at −52 °C, setting the vacuum to 0.100 mBar (LyoQuest −55, Telstar Technologies, Spain). The lyophilized juice was purified from sugars by solid phase extraction (SPE). In detail, the juice was solubilized in distilled water and loaded on a Strata-X 33 μm Polymeric 500 mg/6 mL cartridge (Phenomenex^®^), previously equilibrated in distilled water, then eluted with MeOH 0.1% *v/v* TFA and finally re-lyophilized and stored at −20 °C. Finally, the polyphenolic extract (PPJE) was solubilized in MeOH and filtered through 0.45 μm nylon membrane filters prior to analysis.

### 2.3. Qualitative and Quantitative Profiling of PPJE

Online comprehensive Hydrophilic High performance Liquid Chromatography × Reversed Phase-Ultra High Performance Liquid Chromatography phase (HILIC-HPLC × RP-UHPLC) platform coupled to mass spectrometry was developed for detailed profiling of PPJE. HILIC-HPLC × RP-UHPLC analyses were performed on a Shimadzu Nexera (Shimadzu, Milan, Italy) system (see Supporting Information Section S1).

A Luna^®^ HILIC 150 mm × 2.0 mm, 3.0 μm (200 Å) column (Phenomenex^®^, Castel Maggiore, Bologna, Italy) was employed in ^1^D, whereas a Titan^TM^ C18 50 mm × 3.0 mm, 1.9 μm (80 Å) from Supelco (Bellefonte, PA, USA) was used in the ^2^D. The two dimensions were connected by an ultra-high pressure 10 port-two position switching valve equipped with two trapping columns SecurityGuard^TM^ Ultra C18 2.0 × 4.6 mm, sub-2 μm.

^1^D-LC HILIC separation was carried out with the following parameters: mobile phases, (A) 0.1% CH_3_COOH in MeOH/H_2_O, 95/5 (*v/v*); B) ACN plus 0.1% CH_3_COOH; gradient, 0–1.0 min, 98% B; 1.01–60 min, 98–60% B; 60.01–65.0 min, 60–20% B; flow rate, 100 μL/min. Column oven was set to 25 °C. The dilution flow prior to the trapping was set to 1 mL/min and mobile phase was 0.1% CH_3_COOH in H_2_O (*v/v*). ^2^D-RP mobile phases were A) 0.1% CH_3_COOH in H_2_O (*v/v*), B) ACN plus 0.1% CH_3_COOH. In ^2^D, the gradient was operated in multi-segment shifting mode. Flow rate was set to 2.2 mL/min. Column oven was set to 45 °C. The modulation time was 45 s. A total of 3 μL of PPJE were injected. PDA acquisition was set in the range 190–540 nm and chromatograms were monitored at 280 and 330 nm. 

1D-UHPLC and LC × LC systems were coupled online to IT-TOF instrument equipped with an electrospray source (ESI). MS ionization was operated in negative mode for the polyphenols analysis through 1D-UHPLC and LC × LC platforms, while in positive mode for the anthocyanins analysis by 1D-UPLC-analysis. The identification of bioactive compounds was based on accurate MS and MS/MS spectra, comparison with available standards and in silico MS/MS spectra (Sirius ver. 4.16 and Mass bank of North America, MoNA) (see Supporting Information Section S2). 

Quantitative analysis of bioactive compounds in PPJE was carried out by one dimensional liquid chromatography coupled to mass spectrometry (1D-LC-MS, see Supporting Information Section S3). Ellagic acid and punicalagin were selected as external standard for the quantification of ellagitannins, while pelargonidin 3-O-glucoside was chosen for the quantification of anthocyanin. The calibration curves were obtained in a concentration range of 5–100 μg/mL for ellagic acid, 1–200 μg/mL for pelargonidin 3-O-glucoside, and 50–1000 μg/mL for punicalagin, with five concentration levels and performing triplicate analysis for each level. The amount of the compounds in the sample was expressed as micrograms per milligram of extract. 

### 2.4. Biological Evaluation

#### 2.4.1. Cell Culture 

The intestinal epithelial cell line IEC-6 (CRL-1592), from normal rat intestinal crypts [24], was obtained from the American Type Culture Collection (ATCC, Rockville, MD, USA). IEC-6 cells were routinely maintained in the presence of Dulbecco’s modified Eagle’s medium (DMEM; 4 g/L glucose) containing 10% (*v/v*) fetal bovine serum (FBS), 2 mM L-glutamine, 1.5 g/L NaHCO_3_, and 0.1 U/mL bovine insulin. Cells were grown at 37 °C in a humidified atmosphere of 5% CO_2_ and all experiments were done on cells between passages 16 and 19.

#### 2.4.2. Cell Treatment

IEC-6 cells were plated and, after adhesion, were treated with PPJE (10–1.25 µg/mL) for 1 h and then co-exposed to the tested extract and lipopolysaccharides from *E. coli* (LPS; 10 μg/mL) plus interferon-γ (IFN; 10 U/mL) for different times, based on the mediator to evaluate.

In another series of experiments, in order to evaluate the onconutraceutical potential of PPJE (10–1.25 µg/mL), the tested extract was added to IEC-6 cells for 1 h and then 5-Fluorouracil (5-FU; 10 µg/mL) was also added, for different times, depending on the mediator to evaluate.

#### 2.4.3. Antiproliferative Activity

Antiproliferative activity was evaluated using the colorimetric assay of 3-(4,5-dimethylthiazol-2-yl)-2,5-diphenyltetrazolium bromide (MTT), as formerly reported [25]. IEC-6 cells (2.0 × 10^3^ cells/well) were plated on 96-well plates and allowed to adhere for 24 h at 37 °C in a 5% CO_2_ atmosphere. Thereafter, the medium was substituted with either a new one alone or one containing serial dilutions of PPJE (10–1.25 µg/mL) and incubated for 24 h. In another set of experiments, we assessed the ability of tested extract (10–1.25 µg/mL) to reduce antiproliferative activity of 5-FU, on IEC-6 cells. After cell treatment with PPJE for 1h, as previously reported, 5-FU (10 µg/mL) was added to IEC-6 and incubated for 24 h. After incubation, MTT (5 mg/mL) was then added to IEC-6 cells and 3 h later, cells were lysed with 100 µL of a solution containing 50% (*v/v*) N,N-dimethylformamide, and 20% (*w/v*) sodium dodecyl sulphate (SDS; pH = 4.5). To measure the optical density (OD) in each well a microplate spectrophotometer reader (Titertek Multiskan MCC/340-DASIT, Cornaredo, Milan, Italy) was used. The antiproliferative activity was measured as % dead cells: 100 [(OD treated/OD control) × 100].

#### 2.4.4. TNF-α, IL-6 and IL-1β Determination

TNF-α, IL-6 and IL-1β levels in the supernatant of IEC-6 cells were performed by an Enzyme-Linked Immuno Sorbent Assay (ELISA). IEC-6 cells were plated into 24-well plates (8.0 × 10^4^ cells/well) and allowed to adhere for 24 h. Cells were then treated with PPJE (10–1.25 µg/mL), as indicated, for 18 h. To perform the ELISA, a commercially available kit (e-Bioscience, San Diego, CA, USA) was used, according to the manufacturer’s instructions. Results were expressed as pg/mL of cytokines release as previously reported [26].

#### 2.4.5. Evaluation of COX-2, iNOS, HO-1, NQO1, Bax, Bcl-2, Bcl-xL, Caspase 3, Claudin 1, Occludin, ZO-1, E-cadherin Expression and Nitrotyrosine Formation by Cytofluorimetry

IEC-6 cells were plated into 96-well plates (2.0 × 10^3^ cells/well) and treated with PPJE (10–1.25 µg/mL), as previously indicated for 24 h. For this analysis, IEC-6 cells were then collected and washed with phosphate buffered saline (PBS). First, a fixing solution was added to the IEC-6 cells for 20 min and after the cells were incubated in fix perm solution for a further 30 min. Anti-cyclooxygenase-2 (COX-2; BD Transduction Laboratories, Milan, Italy), anti-inducible nitric oxide synthase (iNOS; BD Transduction Laboratories, Milan, Italy), anti-heme oxygenase-1 (HO-1; Santa Cruz Biotechnologies, Dallas, TX, USA), anti-NAD(P)H dehydrogenase (quinone) 1 (NQO1; Santa Cruz Biotechnologies, Dallas, TX, USA), anti-Bax (Santa Cruz Biotechnologies, Dallas, TX, USA), anti-Bcl-2 (Santa Cruz Biotechnologies, Dallas, TX, USA), anti-Bcl-xL (Thermofisher Scientific, Waltham, MA, USA), anti-caspase 3 (Thermofisher Scientific, Waltham, MA, USA), anti-claudin 1 (Thermofisher Scientific, Waltham, MA, USA), anti-Occludin (Thermofisher Scientific, Waltham, MA, USA), anti-Z-Occludin 1 (ZO-1; Thermofisher Scientific, Waltham, MA, USA), anti-E-cadherin (Cell Signaling Technology, Dellaertweg 9b, The Netherlands) or anti-nitrotyrosine (Merck Millipore, Milan, Italy) antibodies were then added for 1 h. The secondary antibody was added to IEC-6 cells in fixing solution and cell fluorescence was then evaluated by a fluorescence-activated cell sorter (FACSscan; Becton Dickinson, Milan, Italy) and then elaborated by Cell Quest software (version 4; Becton Dickinson, Milan, Italy) [27].

#### 2.4.6. Intracellular ROS Release Evaluation

ROS intracellular levels in IEC-6 were evaluated by the probe 2′,7′-dichlorofluorescein-diacetate (H_2_DCF-DA). Cells were seeded in 24-well plates (8.0 × 10^4^ cells/well) and allowed to adhere for 24 h. Thereafter, cells were incubated with PPJE (10–1.25 µg/mL), as previously indicated for 24 h. In another set of experiments, cells were treated with tested extract (10–1.25 μg/mL) alone for 1 h and then simultaneously exposed to the extract and hydrogen peroxide (H_2_O_2_; 1 mM) for another hour. IEC-6 cells were subsequently collected, washed with PBS, and incubated in PBS with H_2_DCF-DA (10 µM). After an incubation of 15 min at 37 °C cell fluorescence was evaluated with a fluorescence-activated cell sorter (FACSscan; Becton Dickinson, Franklin Lakes, NJ, USA), and was analyzed by CellQuest software (version 4; Becton Dickinson, Milan, Italy) [28].

#### 2.4.7. Analysis of Apoptosis

Hypodiploid DNA was analyzed by propidium iodide (PI) staining through flow cytometry [29]. IEC-6 (3.5 × 10^5^) cells were grown in 24-well plates. After adhesion cells were treated with PPJE (10–1.25 µg/mL) plus 5-FU (10 μg/mL), for further 24 h. Cell culture medium was removed and cells were washed with PBS and suspended in 500 µL of a solution containing 0.1% (*w/v*) sodium citrate and PI (50 μg/mL). The solution was centrifuged and cell pellets were pooled with cell suspension to retain both dead and living cells for analysis and incubated at 4 °C for 30 min in the dark. A Becton Dickinson FACScan flow cytometer using the CellQuest program was used to analyze cell nuclei. 

#### 2.4.8. Scratch Assay for Cellular Migration

Cellular migration was evaluated by wound assay. IEC-6 cells were seeded in 24-well plates (8.0 × 10^4^ cells/well) and incubated for 24 h. After incubation, cells reached 100% confluence and a wound was performed at the center of the monolayer by gently scraping it with a sterile plastic p10 pipette tip to create a wound area. Cells were then washed with PBS and treated with PPJE (5–2.5 µg/mL), in the presence of 5-FU (10 µg/mL) for 24 h. The wounded cells were then placed in a humidified and equilibrated (5% *v/v* CO_2_) incubation chamber of an Integrated Live Cell Workstation Leica AF-6000 LX at 37 °C for 24 h. In order to record cell movements a 10 × phase contrast objective was used, with a frequency of acquisition of 10 min. The migration rate of individual cells was determined, considering the distances covered from the initial time to the selected time points (bar of distance tool, Leica AF software, 2.3.5 build 5379, Leica, Wetzlar, Germany). For each well, at least three different positions were registered and, to measure the migration distances, for each position, at least 10 different cells were randomly selected. To perform the statistical analyses, a GraphPad Prism 5 software (GraphPad, San Diego, CA, USA) was used [30].

### 2.5. Data Analysis

Data are reported as mean ± standard error mean (S.E.M.) of at least three independent experiments, each in triplicate. The statistical analysis has been performed through the analysis of variance test. Bonferroni’s test was used to make multiple comparisons. A *P*-value less than 0.05 was considered as significant.

## 3. Results

### 3.1. Qualitative and Quantitative Profile of Punica granatum L. Extracts

In this work, we applied our previously developed online comprehensive HILIC × RP-MS/MS approach with a trapping modulation interface [31,32] to elucidate in detail the bioactive compounds contained in PPJE.

**Figure 1:**
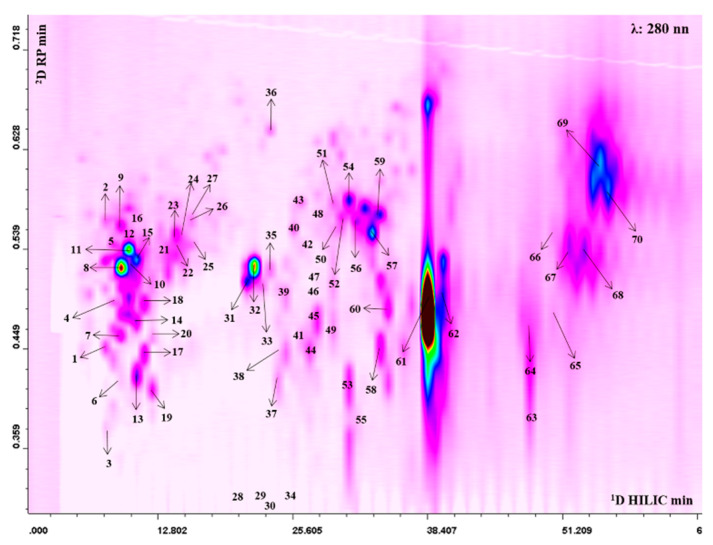
2D Hydrophilic High performance Liquid Chromatography × Reversed Phase-Ultra High Performance Liquid Chromatography phase (HILIC × RP-UHPLC) counter plot (280 nm) with peak assignment of polyphenols isolated from *Punica granatum* L.

The 2D plot extracted at 280 nm with peak assignment relative to the separation obtained with the HILIC × RP approach is shown in Figure 1. The complete list of the bioactive compounds tentatively identified is reported in Table S1. Thanks to high peak capacity and selectivity compared to conventional monodimensional methods the LC × LC platform allowed us to tentatively identify a total of 70 polyphenolic compounds, belonging to different classes: ellagitannins (42), gallotannins (4) hydroxycinnamic acids (10), flavonoids (11) and unknown ellagitannin derivatives (3). Among hydroxycinnamic acids, peak **1** showed a fragmentation pattern consisting of a base peak at *m/z* 193, which assumes the loss of a hexose moiety and the fragment relative to deprotonated aglycone ferulic acid, therefore it was tentatively identified as ferulic acid hexoside. Peaks 2, 5, 12 presented all a strong fragment at *m/z* 353 and a minor fragment at *m/z* 191, therefore they were tentatively proposed as caffeoylquinic acid derivatives. Coumaroyl derivatives were also present. In this regard, peaks 4, 7, 11, 15, 18, 20 were all characterized by the same fragmentation pattern with a fragment at *m/z* 163. In particular, peak 4 and 7, presented an MS/MS fragment at *m/z* 173, relative to the loss of the dehydroquinic acid, thus they were assumed to be coumaroylquinc acids [33]. Among flavonoids, peak 8 at *m/z* 507 showed a fragment ion at *m/z* 345, which suggests the loss of a hexose glycoside moiety, leading to its tentative identification as siringetin-hexoside. The compound 16 showed MS/MS fragment ion at *m/z* 273, again relative to the loss of a hexose moiety together with the deprotonated aglycone phloretin, leading to its annotation as phlorizin. The analytical platform developed led to detection of a higher number of tannins that were eluted in the middle and final part of the 2D plot. Several ellagic and galloyl glycosides were found, which presented a similar MS/MS fragmentation resulting from a hexose [M-H-162]^-^ (28, 29, 30, 32, 33, 34, 35, 38, 42, 48). In addition to ellagic acid hexoside (*m/z* 463) further monoglycosylated ellagic acid derivatives were observed, such as an ellagic acid-pentoside (*m/z* 433; peak 26) and deoxyhexoside (*m/z* 447; peaks 23, 24, 27), all of them showing the typical fragments of ellagic acid at *m/z* 300. Moreover, compounds 44 and 45 produced a precursor ion at *m/z* 799 and fragments at *m/z* 781, relative to the loss of water, and a secondary fragment at *m/z* 479, resulting from the loss of ellagic acid. These compounds could be attributed to granatin A. Compounds 61 and 62 were the most intense in the profile. The alignment with standard retention time, the doubly charged precursor ion at *m/z* 541 and the fragmentation pattern led to their identification as punicalagin and its isomer (Figure S1). Lastly, peaks 64, 68, 69 showed a precursor ion at *m/z* 783 and provided fragment ions at *m/z* 633, deriving from the loss of gallic acid moiety, and at *m/z* 331, which assumes the subsequent loss of ellagic, thus they were proposed as Pedunculagin II isomers (Figure S2a). Moreover, peaks 66 and 70 exhibited the same precursor ion at *m/z* 783, but the loss of water and hexahydroxydiphenic acid produced fragments at *m/z* 765 and *m/z* 481, leading to their tentative identification as pedunculagin I (Figure S2b) [34]. The quantitative analysis showed that pomegranate juice was rich in punicalagin (Table S2). Moreover, we found that in the juice, seven anthocyanins were also present, although not visible in the 2D plot, as they have a typical maximum absorbance between 514 and 540 nm (Figure S3). Cyanidin 3,5-diglucoside and pelargonidin 3-glucoside were the most abundant compounds (Table S3). 

### 3.2. Anti-Inflammatory Activity 

#### 3.2.1. Pomegranate Did not Exert Antiproliferative Activity on IEC-6 Cells

To evaluate the antiproliferative potential of PPJE (10–1.25 µg/mL) in our experimental conditions, IEC-6 cells were treated with extract for 24 h. The results obtained indicated that the tested extract did not have any significant antiproliferative activity on IEC-6 cells (data not shown).

#### 3.2.2. Pomegranate Reduced TNF-α Levels, COX-2 and iNOS Expression, and Nitrotyrosine Formation in LPS + IFN-Stimulated IEC-6

**Figure 2:**
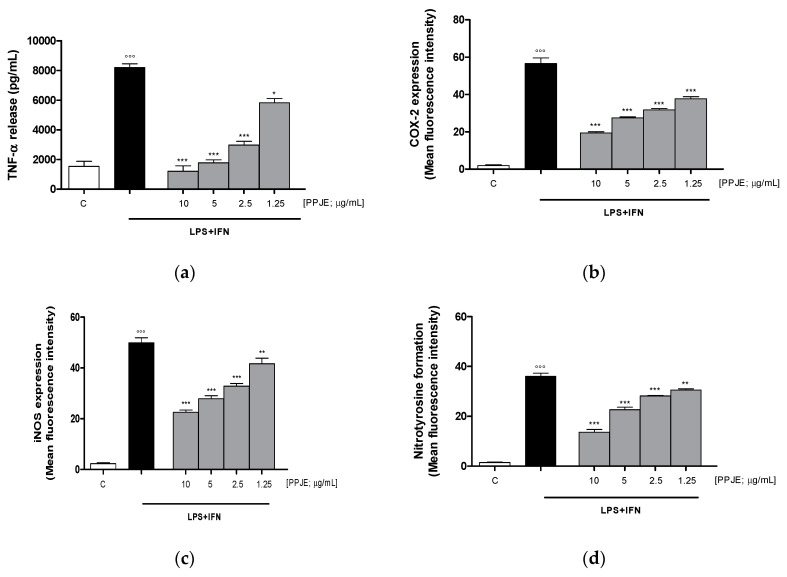
Effect of pomegranate juice extract (PPJE) (10–1.25 µg/mL) on proinflammatory mediators’ inhibition in lipopolysaccharides (LPS) + interferon-γ (IFN) treated IEC-6. TNF-α release (**a**), evaluated by ELISA assay, COX-2 (**b**) and iNOS (**c**) expression and nitrotyrosine formation (**d**), evaluated by the cytofluorimetric technique. Data are expressed as pg/mL or mean of fluorescence intensity. C denotes control group. ***, ** and * denote respectively *p* < 0.001, *p* < 0.01 and *p* < 0.05 vs. LPS + IFN; °°° denotes *p* < 0.001 vs. C.

The effect of pomegranate on TNF-α levels in IEC-6 culture medium was evaluated using an ELISA assay. Our results showed that the tested extract (10–1.25 µg/mL) significantly inhibited TNF-α release, induced by LPS + IFN, at all concentrations tested, in IEC-6 cells (*p* < 0.05 vs. LPS + IFN; Figure 2a). In order to evaluate the anti-inflammatory potential of PPJE, we analyzed the expression of enzymes involved in the inflammatory response, such as COX-2 and iNOS, by flow cytometry. Our results showed that the tested extract (10–1.25 µg/mL) significantly inhibited COX-2 (*p* < 0.001 vs. LPS + IFN; Figure 2b) and iNOS (*p* < 0.01 vs. LPS + IFN; Figure 2c) expression in IEC-6 cells at all tested concentrations.

In this study, we also evaluated the ability of PPJE to influence nitrotyrosine formation, a product of tyrosine nitration mediated by reactive nitrogen species, such as peroxynitrite anion and nitrogen dioxide. Nitrotyrosine is identified as a marker of cell damage, inflammation as well as of nitric oxide production. Our results demonstrated that PPJE (10–1.25 µg/mL) significantly inhibited nitrotyrosine formation at all the tested concentrations, evaluated by flow cytometry (*p* < 0.01 vs. LPS + IFN; Figure 2d). 

#### 3.2.3. Pomegranate Reduced Intracellular ROS Release, Increased HO-1, NQO1, Claudin-1 and ZO-1 Expression in LPS + IFN-Stimulated IEC-6

**Figure 3:**
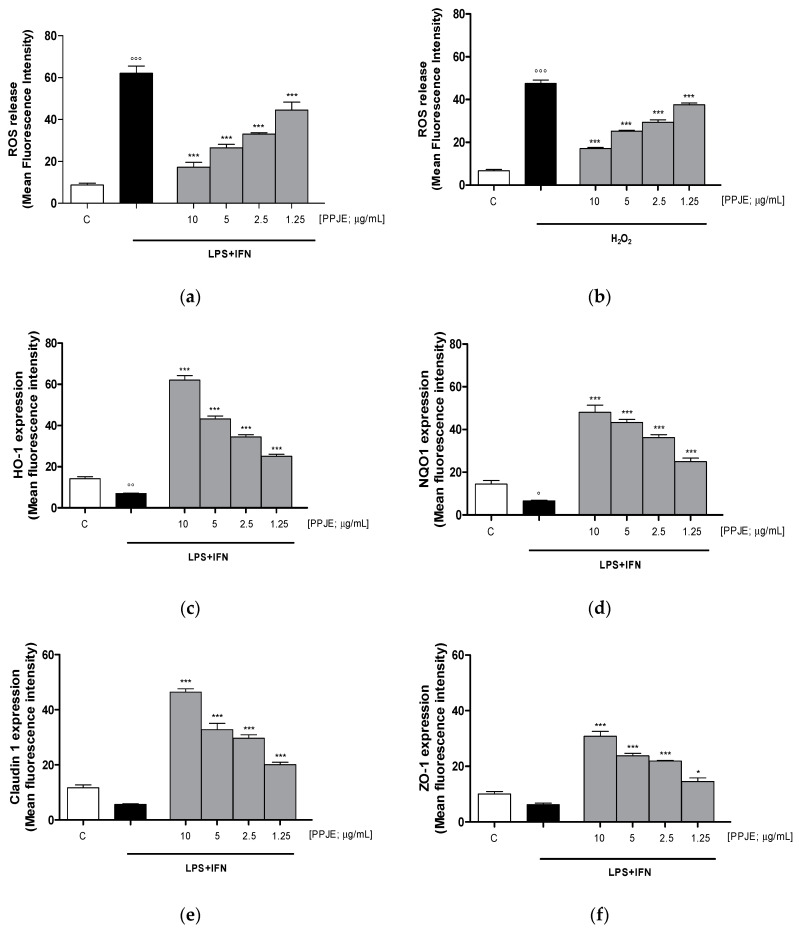
Effect of PPJE (10–1.25 µg/mL) on the inhibition of intracellular ROS release in LPS + IFN (**a**) and in H_2_O_2_ (**b**)–stimulated IEC-6, detected by 2′,7′ dichlorofluorescein-diacetate (H_2_DCF-DA). Effect of PPJE (10–1.25 µg/mL) in increasing the antioxidant proteins HO-1 (**c**) and NQO1 (**d**). In the lower panels the effect of PPJE on the expression of the tight junction (TJ) proteins Claudin-1 (**e**) and ZO-1 (**f**), detected by flow cytometry, was reported. Data are expressed as mean fluorescence intensity. C denotes control group. *** and * denote respectively *p* < 0.001 and *p* < 0.05 vs. LPS + IFN or H_2_O_2_; °°°, °° and ° denote respectively *p* < 0.001, *p* < 0.01 and *p* < 0.05 vs. C.

Oxidative stress is due to an imbalance between pro- and antioxidant cellular systems, thus, we firstly evaluated the effect of the tested extract on intracellular ROS production in LPS + IFN-stimulated IEC-6 cells. Our results indicated that pomegranate extract (10–1.25 µg/mL) significantly reduced, at all tested concentrations, intracellular ROS release in IEC-6 during inflammatory response (*p* < 0.001 vs. LPS + IFN; Figure 3a).

In order to evaluate the effect of PPJE in the presence of a pro-oxidant stimulus, in another set of experiments, IEC-6 cells were treated with H_2_O_2_ (1 mM). Our results showed that the tested extract significantly reduced ROS release in H_2_O_2_-treated IEC-6 (*p* < 0.001 vs. H_2_O_2_; Figure 3b).

The effect of PPJE on the antioxidant cellular response was evaluated on the cytoprotective enzymes, such as HO-1 and NQO1, determined by flow cytometry. Our results demonstrated that pomegranate extract (10–1.25 µg/mL) significantly increased HO-1 and NQO-1 expression (*p* < 0.001 vs. LPS + IFN; Figure 3c,d).

The anti-inflammatory potential of the extract during intestinal inflammation was also demonstrated by its ability to modulate the expression of tight junction proteins, such as Claudin-1 and ZO-1, in which reduction during inflammation is responsible for the increased permeability of the intestinal epithelium. Our results showed that pomegranate extract (10–1.25 µg/mL) significantly increased Claudin-1 and ZO-1 expression, at all tested concentrations, compared to LPS + IFN-stimulated IEC-6 (*p* < 0.05 vs. LPS + IFN; Figure 3e,f).

### 3.3. Onconutraceutical Potential

#### 3.3.1. Pomegranate Reduced ROS Release and Increased HO-1 and NQO1 Expression in 5-FU-treated IEC-6

**Figure 4:**
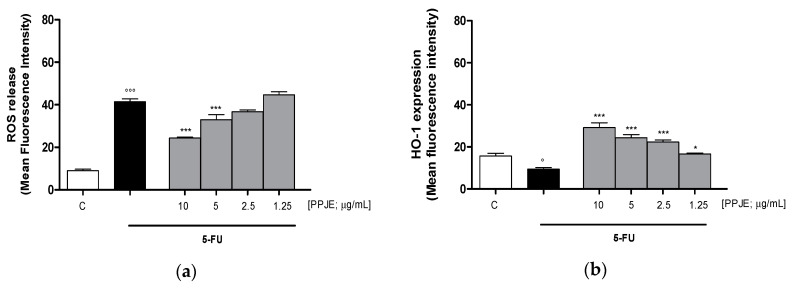

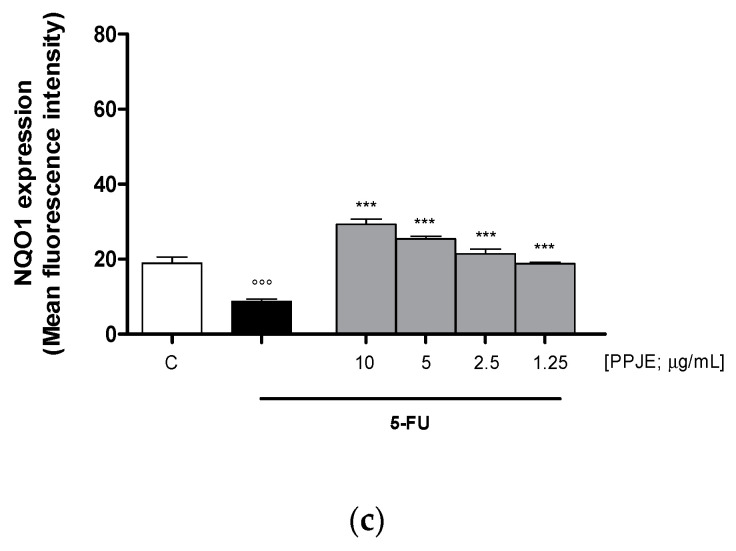
Effect of PPJE (10–1.25 µg/mL) on the inhibition of intracellular ROS release (**a**), detected by 2′,7′ dichlorofluorescein-diacetate (H_2_DCF-DA), and on the increased expression of the antioxidant proteins HO-1 (**b**) and NQO1 (**c**), detected by flow cytometry, on 5-FU-treated IEC-6. Data are expressed as mean fluorescence intensity. C denotes control group. *** and * denote respectively *p* < 0.001 and *p* < 0.05 vs. 5-FU; °°° and ° denote respectively *p* < 0.001 and *p* < 0.05 vs. C.

Oncology therapies with 5-FU are associated with an excessive ROS release, mainly responsible for the establishment of oxidative stress. To assess the antioxidant potential of PPJE also in 5-FU-treated IEC-6 cells, intracellular ROS release and cytoprotective enzymes expression were evaluated. PPJE (10–1.25 µg/mL) significantly reduced ROS release compared to 5-FU (10 µg/mL) treated cells (*p* < 0.001 vs. 5-FU; Figure 4a) and significantly increased HO-1 (*p* < 0.05 vs. 5-FU; Figure 4b) and NQO1 (*p* < 0.001 vs. 5-FU; Figure 4c) expression, compared to IEC-6 treated with 5-FU alone. These results further highlight the antioxidant and onconutraceutical potential of PPJE on IECs also during 5-FU based chemotherapy.

#### 3.3.2. Pomegranate Reduced Cytokines Levels, COX-2 and iNOS Expression and Nitrotyrosine Formation in 5-FU-Treated IEC-6

**Figure 5:**
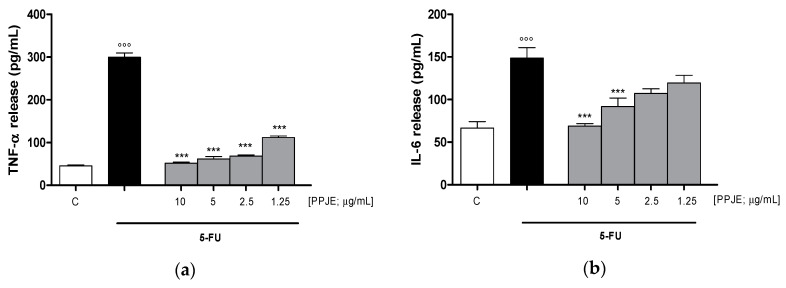

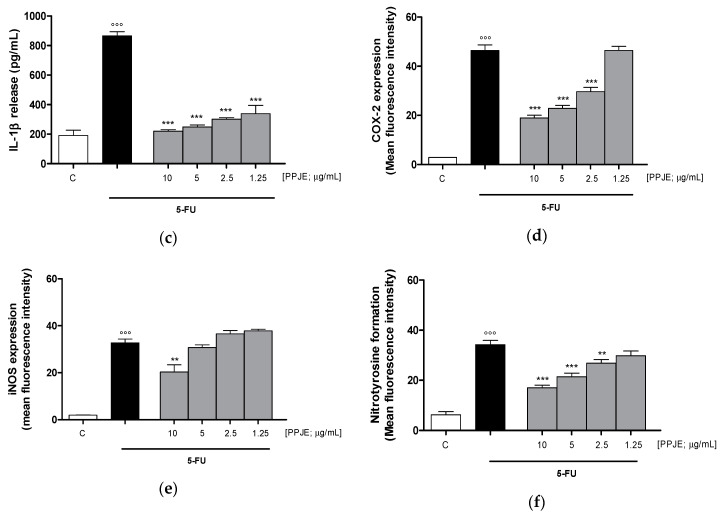
Effect of PPJE (10–1.25 µg/mL) on proinflammatory mediators’ inhibition in 5FU-treated IEC-6. TNF-α (**a**), IL-6 (**b**) and IL-1β (**c**)were detected by ELISA assay. Effect of PPJE (10–1.25 µg/mL) on COX-2 (**d**) and iNOS (**e**) expression, and nitrotyrosine formation (**f**), detected by flow cytometry. Data are expressed as pg/mL of cytokines release or as mean fluorescence intensity. *** and ** denote respectively *p* < 0.001 and *p* < 0.01 vs. 5-FU; °°° denotes *p* < 0.001 vs. C.

Considering the inhibitory effect on TNF-α release following the treatment with pomegranate juice under inflammatory conditions, we also assessed the activity of PPJE on cytokines release in IEC-6 cells, treated with 5-FU. Our results indicated that pomegranate extract (10–1.25 µg/mL) significantly inhibited TNF- α (*p* < 0.001 vs. 5-FU; Figure 5a), IL-6 (*p* < 0.001 vs. 5-FU; Figure 5b) and IL-1β (*p* < 0.001 vs. 5-FU; Figure 5c) release, evaluated by ELISA assay. The inhibition of inflammatory enzymes, such as COX-2, for PPJE three highest concentrations, and iNOS (*p* < 0.01 vs. 5-FU; Figure 5d,e), for the highest PPJE concentration, was also observed in 5-FU-treated IEC-6 cells. Moreover, PPJE also reduced nitrotyrosine formation, an important marker of nitrosative stress, compared to IEC-6 treated with 5-FU alone at the three highest concentrations (*p* < 0.01 vs. 5-FU; Figure 5f). These results further confirmed the anti-inflammatory, antioxidant and onconutraceutical potential of this extract in IECs.

#### 3.3.3. Pomegranate Reduced 5-FU-Induced Apoptosis in IEC-6

**Figure 6:**
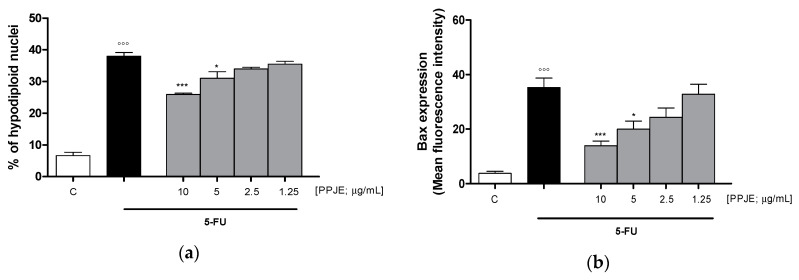

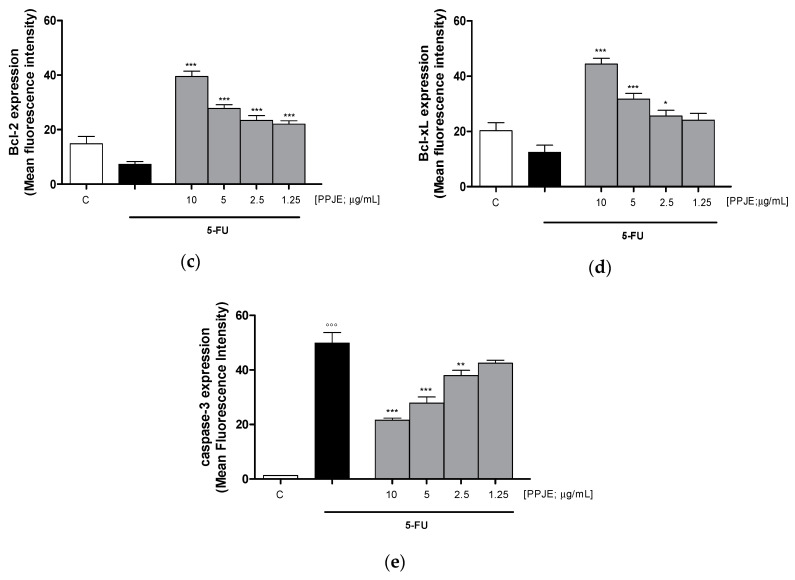
Effect of PPJE extract (10–1.25 µg/mL) on 5-FU-treated IEC-6in reducing apoptosis (**a**), detected by propidium iodide (PI) assay, on Bax (**b**), Bcl-2 (**c**), Bcl-xL (**d**) and caspase-3 (**e**) expression, detected by flow cytometry. Data are expressed as % of hypodiploid nuclei or mean fluorescence intensity. C denotes control group. ***, ** and * denote respectively *p* < 0.001, *p* < 0.01 and *p* < 0.05 vs. 5-FU; °°° denotes *p* < 0.001 vs. C.

To evaluate the ability of PPJE to affect 5-FU-induced apoptosis in IEC-6 cells, we analyzed PI stained hypodiploid nuclei. Our results indicated that PPJE (10–1.25 µg/mL) reduced apoptosis induced by 5-FU (10 µg/mL) significantly at the highest concentrations tested (*p* < 0.05 vs. 5-FU; Figure 6a). Moreover, we observed that PPJE significantly reduced the expression of Bax, a pro-apoptotic protein (*p* < 0.05 vs. 5-FU; Figure 6b) and increased the expression of Bcl-2 and Bcl-xL, two anti-apoptotic proteins (*p* < 0.05 vs. 5-FU; Figure 6c,d).

The anti-apoptotic potential of pomegranate juice was also supported by its ability to reduce caspase-3 expression, compared to 5-FU-treated IEC-6 (*p* < 0.01 vs. 5-FU; Figure 6e).

#### 3.3.4. Pomegranate Juice Promoted Wound Repair and Adhesion Proteins Expression in 5-FU-Treated IEC-6

**Figure 7:**
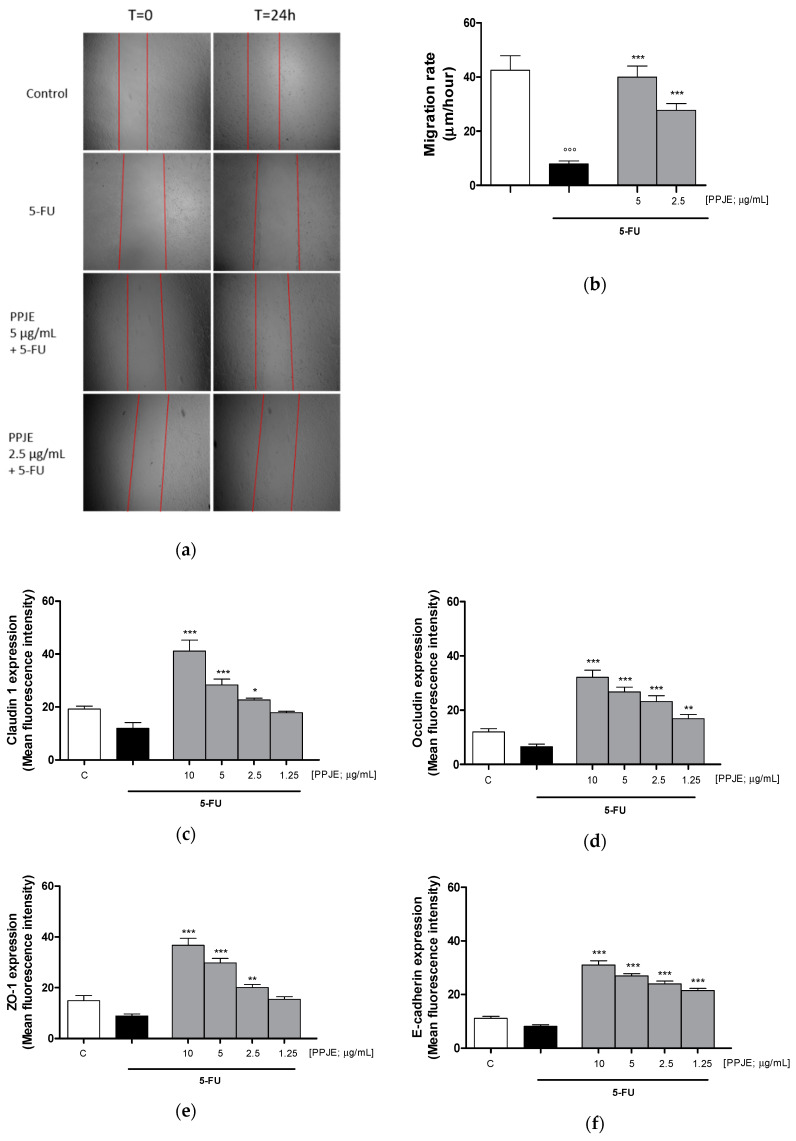
Effect of PPJE (5–2.5 µg/mL) on wound repair after a mechanical scratch in IEC-6 treated with 5-FU (10 µg/mL); Bar = 150 μm (**a**), and the quantitative analysis expressed as IEC-6 migration rate after 24 h (**b**). Effect of PPJE (10–1.25 µg/mL) in increasing Claudin-1 (**c**), Occludin (**d**), ZO-1 (**e**) and E-cadherin (**f**) expression, detected by flow cytometry, on 5-FU-treated IEC-6. Values are expressed as migration rate (μm/hour) or mean fluorescence intensity. C denotes control group. ***, ** and * denote respectively *p* < 0.001, *p* < 0.01 and *p* < 0.05 vs. 5-FU; °°° denotes *p* < 0.001 vs. C.

Wound repair at the intestinal level is extremely important for healing of ulcers induced by chemotherapy treatments. For this reason, we analyzed the ability of PPJE to modulate cell migration speed by scraping the monolayer of IEC-6 cells treated with 5-FU (10 µg/mL). We observed that pomegranate (5–2.5 µg/mL) significantly enhanced cell migration speed compared to 5-FU-treated cells (*p* < 0.01 vs. 5-FU; Figure 7a,b). Adhesion proteins play a key role during tissue repair processes. Pomegranate increased Claudin-1 (*p* < 0.05 vs. 5-FU; Figure 7c), Occludin (*p* < 0.01 vs. 5-FU; Figure 7d), ZO-1 (*p* < 0.01 vs. 5-FU; Figure 7e) and E-cadherin (*p* < 0.001 vs. 5-FU; Figure 7f) expression in 5-FU-treated IEC-6.

## 4. Discussion

The gut inflammatory response is coordinated by a complex network of mediators released from cells underlying the lamina propria and intestinal epithelial cells (IECs). IECs can be therefore considered as integral components of the immune system by taking an active part in both innate and acquired immunity through the secretion of inflammatory and oxidative stress mediators. While inflammation is a fundamental defense strategy against aggressors, its exacerbation due to exaggerated and/or persistent stimuli could lead to a chronic response, increasing the risk of developing severe pathologies [35]. Inflammatory pathways are also triggered by radiotherapy and chemotherapy treatments, leading to severe side effects, such as gastrointestinal mucositis associated with a severe impairment of nutritional conditions and a reduction/interruption of anticancer treatment, with an increased risk of overall complication and mortality [36]. Therefore, control of inflammatory and oxidative stress responses is of primary importance to avoid deleterious pathological conditions and to counteract drugs’ side effects. Polyphenols-rich foods, thanks mainly to their anti-inflammatory and antioxidant potential, are increasingly being used in combination with chemotherapy protocols to improve their efficacy and reduce adverse effects [37,38,39,40,41].

*Punica granatum* L., better known as pomegranate, is an ancient fruit widely consumed as fresh fruit and juice. It is increasingly used in cosmetic, pharmaceutical and nutraceutical fields for its antioxidant, anti-inflammatory, anti-microbial and antiproliferative properties, related to the presence of several bioactive phytochemicals of very different size and polarity [42]. In particular, pomegranate peel extract has attracted much attention for colorectal cancer preventive properties related to the anti-inflammatory, pro-apoptotic and antiproliferative activity of the bioactive tannins both in vitro and in vivo [43,44,45]. However, the potential role of pomegranate polyphenols present in juice extract in current chemotherapeutic anticancer protocols to prevent and alleviate intestinal injury induced by 5-fluorouracil (5-FU) on non-tumorigenic intestinal epithelial cells has been scarcely reported.

In this study, we evaluated the effect of pomegranate juice on inflammatory and oxidative stress mediators in IEC-cells both during inflammation response and following 5-FU treatment of the cells.

An accurate characterization of the phytocomplex is mandatory to understand the mechanisms of action of different compounds. Online comprehensive two dimensional Liquid Chromatography (LC × LC) takes advantage of two different separation methods, resulting in higher selectivity compared to 1D-LC and is well suited for complex matrices characterization [46]. In the present work, given the sample complexity, mainly due to the high content in hydrolyzable tannins of different size and weight, we applied a previously developed LC × LC-MS/MS platform to the detailed and multiclass accurate profiling of polyphenols in pomegranate juice with high nutraceutical value, without any particular extraction process or a priori fractionation. The modulation approach used overcomes the solvent mismatch deriving from the coupling of HILIC and RP separations, without losing sensitivity and resolution [32]. The combination of two different separation methods, allows a class-based separation, resulting in easier identification in the further combination with mass spectrometry detection. In this regard, the number of detected compounds, in particular of phenolic acids and ellagitannins, was higher compared to previous profiling analyses on pomegranate fruits carried out by LC-MS techniques [34,47,48,49].

After analytical characterization of the main polyphenolic compounds present in the pomegranate juice, their biological evaluation revealed that they were able to reduce the inflammatory and oxidative stress response triggered by both LPS and 5-FU, and to stimulate wound repair in the non-tumorigenic cell line IEC-6. One of the major cytokines playing a pivotal role in intestinal inflammation is TNF-α. In IECs, this cytokine is significantly upregulated by proinflammatory stimuli [50]. In addition, proinflammatory enzymes, such as iNOS or COX-2, are also elevated as a consequence of intestinal inflammation. They contribute both to amplification of the inflammatory response (also via TNF-α) and to oxidative stress [51,52]. In our experiments, pomegranate juice significantly inhibited TNF-α release, as well as COX-2 and iNOS expression. These data are in accordance with previous findings indicating the ability of a pomegranate beverage to suppress the expression of proinflammatory cytokines, such as TNF-α, and inflammation-related proteins COX-2 and iNOS, evaluated at their mRNA levels in a rat colitis model [53]. Nitric oxide (NO), produced in higher amounts during intestinal inflammation [54], is able to react with ROS leading to nitrotyrosine formation, a marker of nitrosative stress. Nitrotyrosine is considered to be an iNOS-dependent marker [55]. Our data indicate that pomegranate juice is also able to significantly reduce nitrotyrosine formation at all tested concentrations in IEC-6 during intestinal inflammation. The antioxidant effect of PPJE was then investigated further. In our experimental system, PPJE also showed a strong antioxidant effect reducing ROS release, both in LPS- and in H_2_O_2_- treated cells, and increasing the Nrf-2 mediated antioxidant response, as indicated by the significant increase of the cytoprotective enzymes HO-1 and NQO1 in IEC-6 during inflammation. These results agree with previous data reporting the antioxidant potential of punicalagin, an abundant ellagitannin present in pomegranate juice, that protects IEC-6 cells against heat stress-induced gastrointestinal injury by upregulating the expression of HO-1 via a mechanism that also involves Nrf2 translocation [56]. Moreover, pomegranate juice also increased tight junctions’ proteins involved in the breakdown of the intestinal barrier during inflammatory response, such as Claudin-1 and ZO-1, a result consistent with previous evidence reporting the pomegranate’s ability to prevent the changes in gut adherens junction proteins in binge alcohol-exposed rats [57].

Thus, considering the possible role of the mediators evaluated here also in intestinal mucositis associated with chemotherapy, the effect of PPJE was also studied in IECs treated with 5-FU. Previous studies already suggested a protective effect of pomegranate juice in intestinal mucositis, at the level of intestinal epithelial cells [58], but the novelty of this study rests, in particular, on the use of a non-tumorigenic cell line and in the evaluation of parameters potentially involved in the five-phase model of mucositis development, proposed by Sonis [8]. In brief, the stages of this model include the phases of (1) initiation, (2) upregulation, (3) signal amplification, (4) ulceration and (5) healing. Whatever the target tissue, generation of oxidative stress and ROS by chemotherapeutic agents or radiation appears to be a primary event in most pathways leading to the mucositis initiation phase [59]. In accordance with this model, in our experiments 5-FU treatment induced oxidative stress in IEC-6 both increasing ROS release and reducing the cytoprotective enzymes HO-1 and NQO1. Pomegranate juice reduced oxidative stress both by reducing ROS release and by increasing the antioxidant response through HO-1 and NQO1 increase. ROS activation stimulates transcription factors that are considered the hallmark of the initiation phase of mucositis leading to the subsequent other biologic events [8]. During the second phase, proinflammatory factors, such as TNF-α, COX-2 and iNOS, contribute to tissue injury and apoptosis, further amplificated in the third phase. Our results indicate that pomegranate juice reduced these proinflammatory mediators and also nitrotyrosine formation in IEC-6 cells treated with 5-FU. Exposure of IEC-6 cells to 5-FU greatly reduced IEC-6 cell viability when compared to non-damaged control cells. PPJE is also able to reduce apoptosis in 5-FU-treated cells. The normal balance between pro-apoptotic members of the family (such as Bax) and anti-apoptotic components (for example Bcl-xL) might be disrupted by the administration of mucotoxic therapy. PPJE was able to reduce Bax expression and to increase anti-apoptotic factors, such as Bcl-2 and Bcl-xL. The reduction of apoptotic rate was also associated with a significant reduction in caspase-3 expression. These results agree with previous data reporting the ability of ellagitannins from pomegranate to ameliorate intestinal mucositis and reduce apoptosis of intestinal cells induced by 5-FU in rats [58]. In most cases, mucositis is an acute phenomenon that is self-resolving once cancer therapy ends. Rapid repair of the ulcer site by migration and covering of the epithelial cells is an important process during the restoration of ulcer sites. Tight junction (TJs) family members, including occludin, claudins, zonula occludens (ZOs), in addition to regulation of intestinal barrier function, control epithelial homeostasis and these proteins are also expressed in the intestinal crypt-luminal axis. The expression of these proteins, balanced in physiologic conditions, is altered during intestinal inflammation, like mucositis [60]. In conditions of intestinal epithelium injury, as during mucositis, detachment of the epithelium from the basement membrane and separation of adjacent epithelial cells due to dysregulation and loss of TJs occurs. Closure of the tight junction after acute intestinal injury is a predominant aspect in restoring barrier function and homeostatic functioning [61]. In particular, down regulation of claudin-1, occludin and ZO-1 contributes to increased intestinal permeability that results in cell polarity loss, [62]. In addition, oxidative-stress-induced intestinal permeability is thought to be mediated through the tyrosine phosphorylation of occludin and the redistribution of occludin, ZO-1 and E-cadherin, that could delocalize/disassemble from the TJs [63]. Our results showed that PPJE is able to increase cellular migration rate in IEC-6, in a scratch model, with respect to 5-FU alone. This effect is associated with the significant effect, at almost all tested concentrations, on adhesion proteins primarily involved in the intestinal barrier function such as Claudin-1, Occludin, ZO-1 and E-cadherin.

## 5. Conclusions

The results reported here highlight the dual role of pomegranate juice in the onconutraceutic field. In fact, pomegranate juice, through its ability to reduce inflammatory and oxidative stress in intestinal epithelial cells, could play a role both in reducing intestinal inflammation and in the onconutraceutical field as potential treatment for 5-FU-induced mucositis. Thus, the combination of pomegranate juice with chemotherapy appears to be a promising strategy for protecting against inflammatory and oxidative injury at the intestinal level, as well as intestinal damage observed in 5-FU-induced toxicity.

## Supplementary Materials

The following are available online at https://www.mdpi.com/2076-3921/9/8/699/s1, Section S1: 1D-LC and LC × LC instrumentation; Section S2: Ion trap-Time of flight (IT-TOF) parameters; Section S3: Chromatographic parameters for 1D-LC analyses; Section S4: Effect of pomegranate on 5-FU-induced antiproliferative activity; Figure S1: MS (top) and MS/MS (bottom) spectra of Punicalagin isolated from *Punica granatum* L.; Figure S2: MS (top) and MS/MS (bottom) spectra of Pedunculagin (Bis-HHDP-hexoside) II (a) and Pedunculagin (Bis-HHDP-hexoside) I (b) isolated from *Punica granatum* L.; Figure S3: Chromatogram profile of anthocyanins (λ: 520 nm) isolated from *Punica granatum* L.; Table S1: HILIC-HPLC × RP- UHPLC-PDA-ESI-IT-TOF identification of polyphenols isolated from *Punica granatum* L. fruits; Table S2: Quantitative profile of the main polyphenols isolated from *Punica granatum* L. fruits by HPLC-PDA; Table S3: RP-UHPLC-UV-ESI^+^ identification and quantitation of anthocyanins isolated from *Punica granatum* L. fruits.
